# Chladni figures reduce ohmic losses in alkaline electrolysis

**DOI:** 10.1016/j.ultsonch.2026.107833

**Published:** 2026-03-22

**Authors:** Vid Agrež, Zeinab Heidary, Manolis Gavaises, Rok Petkovšek, Claus-Dieter Ohl

**Affiliations:** a Faculty of Natural Sciences, Institute for Physics, Otto-von-Guericke-University Magdeburg, Universitätsplatz 2, 39106 Magdeburg, Germany; b Faculty of Mechanical Engineering, University of Ljubljana, Aškerčeva 6, SI-1000 Ljubljana, Slovenia; c Faculty of Process Engineering, Otto-von-Guericke-University Magdeburg, Universitätsplatz 2, 39106 Magdeburg, Germany; d School of Science & Technology, City, University of London, Northampton Square, EC1V 0HB London, United Kingdom

**Keywords:** Alkaline electrolysis, Bjerknes force, Hydrogen generation, Chladni figures

## Abstract

An method for hydrogen bubble removal through ultrasonic excitation applied directly through the cathode is presented in this work. High-speed imaging, pressure field measurements, and overvoltage monitoring were employed to characterize how cathode vibration in the 100 kHz range influences electrolytic efficiency. Upon the start of the vibration, hydrogen bubbles detached and migrated across the surface, forming distinct spatial patterns corresponding to the electrode’s vibrational modes and resembling Chladni figures. The bubble migration is dominated by the primary Bjerknes force arising from acoustic pressure gradients. This is confirmed by the Keller-Miksis model which predicts the observed movement of the bubbles to the pressure nodes. The work demonstrates that electrode vibrations significantly reduced ohmic losses, with overvoltage dropping 20% within 100 ms during a 2 s activation period. The voltage required 7 s to return to initial values after deactivation, indicating that pulsed vibration strategies can achieve superior efficiency compared to continuous running setups.

## 1 Introduction

Green hydrogen produced from renewable energy sources [1] has great potential as a clean energy storage solution due to its high energy density of 120 MJ∙kg^−1^ [2]. The efficiency of converting electricity into hydrogen and vice versa is of crucial importance for further improvement of the technology. The workhorse of industrial electrolyzers is the alkaline water electrolysis, having a long lifetime of the electrolysis cell, while accessible metals such as nickel or iron can be used as electrodes [3]. Current densities between 200 and 500 mA∙cm^−2^ and a potential between 1.8 V and 2.5 V [3] are usually required to operate such cells, while the hydrogen production efficiency achievable in industrial grade systems is around 70 % [4].

The efficiency of the process is strongly affected by the rates for formation and detachment of the gaseous products on the electrodes, i.e. hydrogen and oxygen bubbles [5]. In absence of an external flow, hydrogen bubbles that form on the electrode surface, grow and only detach once the buoyancy force prevails [6]. While increasing the current initially enhances bubble production, the bubbles also impede ion transport to the electrodes [7] and reduce the active surface area [8]. Furthermore, bubbles both on the electrodes and within the electrolyte increase ohmic resistance by blocking ion pathways [9], [10], [11]. It is therefore advantageous to remove hydrogen and oxygen bubbles from the cathode and anode as rapidly as possible.

Factors influencing bubble detachment include electrolyte properties [12], current densities, and the fluid dynamics [13] within the electrolytic cell. The various bubble interactions such as bubble coalescence [14], [15], [16] that may cause or accelerate the bubble detachment process, must also be taken into account. In addition, already detached bubbles can influence the early detachment of new bubbles [17] if the electrodes are arranged vertically. We briefly report on measures taken to reduce the ohmic resistance through passive or active bubble detachment methods. Active methods, here defined as techniques that require an external energy input as opposed to passive approaches relying for example solely on surface properties.

Structuring the electrode surface [10] is the most common passive method for early bubble removal. Structuring the electrode with a combination of hydrophobic and gas-forming areas forces the bubbles to migrate towards the hydrophobic areas [11]. Superwetting electrodes have been shown to increase the energy efficiency due to early detachment bubbles [18].

Active methods utilize either flows [19], magnetic fields [20], [21], or acoustic waves. In flow-based removal, bubbles are detached either by increasing the flow and thus the drag or through a centripetal force as a result of electrode rotation. It has also been shown that a convection in the electrolyte induced by a strong magnetic field as a result of the Lorentz force enhances the current density for ferromagnetic and paramagnetic electrodes [21]. Acoustic early detachment methods commonly rely on a sound source that is fed from outside electrolyte, for example using an ultrasonic horn [22], [23], [24], [25]. Yet, two arrangements were reported where the electrode served as the sound source itself, either through high frequency driving using surface acoustic waves or by being connected to a rod that is oscillating at low frequency [26], [27].

In experiments where the acoustic field is introduced from outside the electrolyte, wire electrodes have the advantage to provide a clear view on the bubbles exposed to the ultrasound field. It was reported that at a driving frequency of 38 kHz the bubbles become smaller and plumes formed that accumulate in the standing sound field [22]. The findings have been confirmed at a lower frequency [28]. An increased production efficiency of 4.5 % and energy efficiency of 1.3 % was reported for round nickel electrodes sonicated with 20 kHz ultrasound in a 0.1 M NaOH electrolyte [23]. For square electrode surfaces sonicated also at 20 kHz it was revealed that the bubbles migrated from their initial nucleation sites and coalesced before detachment [24]. Besides the positive effects it was shown that intense ultrasound (here 2 min, 60 W/cm^2^, 20 kHz) may lead to inertial cavitation and electrode erosion [25]. The use of an external ultrasound source such as the immersion ultrasonic horn also requires a sufficiently large liquid gap for the ultrasound to cover the electrode stack. An electrode that is also the source of the ultrasound can be advantageous in this respect. This was realized by vibrating (excitation frequencies up to 33.3 kHz) the electrode holders outside of the electrolytic cell [26]. The removal of the bubbles was observed resulting in a potential drop of 0.14 V.

In this work, we investigate an active method to remove hydrogen bubbles by applying ultrasound through an electrode itself and show how the vibrating electrode surface affects the efficiency of hydrogen generation and the behavior of the bubbles on the electrode. In contrast to previous works, we focus on the mechanisms leading to the attached hydrogen bubble removal from the electrode surface into the surrounding liquid by operating the electrode at one of its structural resonance frequencies. For this purpose, a nickel plate electrode (30 mm x 30 mm) bonded to a piezoelectric transducer of 20 mm diameter is operated in alkaline water (0.1 M NaoH). After switching on the vibration, a bubble pattern resembling Chladni figures is observed [29]. It is interesting to mention that a manipulation of Chladni patterns has been used previously to control the motion of solid objects [30]. In the context of bubbles in liquid environment we are not aware of utilizing this approach.

We will compare the overpotential with the coverage of the electrode surface with bubbles. Further, driving the vibration in a pulsed manner the motion of many bubbles from the start of the excitation is studied and linked to the Bjerknes forces [31] and their resonance frequencies [32]. It is observed that bubbles close to each other start to vibrate and move towards each other due to the secondary Bjerknes force [33], [34]. Additionally, the primary Bjerknes force [31] drives them towards the pressure nodes.

## 2 Experimental approach

**Fig. 1:**
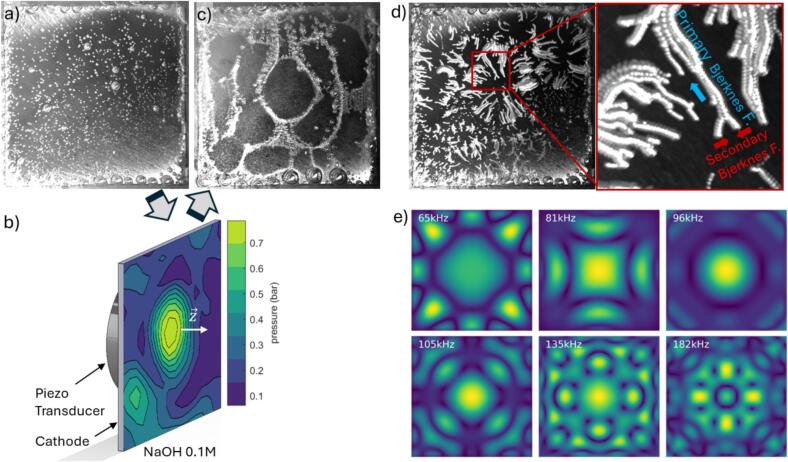
Hydrogen bubble removal mechanism based on an cathode vibration: a) Cathode covered by the hydrogen bubbles, b) 3D model of a vibrating 30 mm x 30 mm cathode on which measured pressure is superimposed, c) Chladni figures observed on a vibrating cathode surface, d) Bubble paths after the application of the vibration with the red inset showing two types of movement, e) Modelled Chladni figures at resonant frequencies for the ideal piezo cathode composite.

To test the effect of vibrating the electrode itself to improve the electrolysis efficiency, we used a nickel electrode driven by a piezoelectric element bonded to its back (Fig. 1). The electrode was submerged in an alkaline electrolyte 0.1 M NaOH and imaged with the high-speed camera. The detailed experimental setup is described in section 2.1. By applying a constant current to the electrode, the hydrogen bubbles are first formed and remained attached on its surface (Fig. 1a). Upon driving the piezo element at 96 kHz with a power amplifier (T&C AG1020) the electrode responds with vibrations and radiates acoustic waves. The resulting pressure distribution just above the surface has been measured with a hydrophone (Reson TC4038) and is depicted in Fig. 1b. The surface vibrations causes the bubbles to detach and migrate. As they move, they begin to concentrate along distinct regions (Fig. 1c). Tracing the bubble movement shows two distinct movement types (Fig. 1d). The first one (marked in red) shows bubbles moving to one another, which we explain with the secondary Bjerknes force, while the second type of movement is towards certain regions on the plate and is caused by the primary Bjerknes force (marked in blue). After the movement to the nodes the hydrogen bubbles form a pattern that resemble vibrational modes of a flat plate. The modes for several plate resonances for an ideally coupled piezo element to the cathode surface are shown in Fig. 1e while the model is described in section 2.2. These patterns resemble Chladni figures that appear when a thin plate covered with sand is brought into vibration with a violin bow. The vibrating surface rearranges the sand particles, accumulating sand at locations of lowest vibration amplitude. These locations are the nodal points lines of the plate’s vibrational modes. Here, also the primary Bjerknes acting on the bubbles vanishes.

### 2.1 Experimental setup

**Fig. 2:**
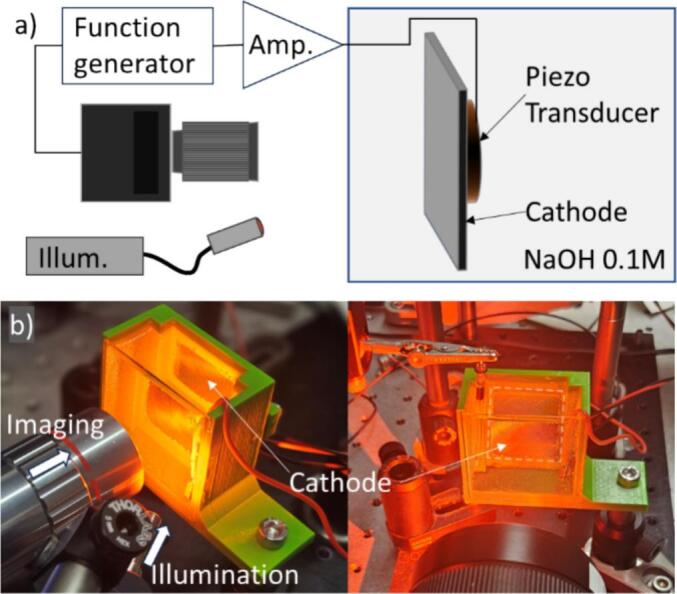
Experimental setup: a) showing the function generator triggering the high-speed camera and providing the driving signal through the amplifier for the piezo transducer. b) showing a cuvette with the cathode surface positioned at the distant wall front illuminated and imaged trough a 5x and 2x magnification lens.

The electrolytic cell consists of a 3D-printed cuvette (50 × 50 × 25 mm^3^) with a glass observation window (50 × 50 mm^2^) positioned opposite the cathode (Fig. 2). A nickel cathode (30 × 30 × 1 mm^3^) is mounted on the back wall and bonded to a piezoelectric transducer with a diameter of 20 mm. The piezo transducer was driven at 96 kHz using a power amplifier (AG series, T&C Power Conversion). A separate nickel anode is positioned laterally within the cuvette. The cuvette is filled with an alkaline electrolyte (0.1 M NaOH).

High-speed imaging of the cathode surface is performed using a Photron Fastcam Mini AX200 camera either with an 5x (Optosigma) or a 2x (Lowa macro) magnification lens. Front illumination is provided by an LED light source (Lightsource.tech LH-HP1, red) operating in continuous mode. The chosen high-intensity illumination is compatible with the short exposure times required at high frame rates used to capture bubble dynamics shown in Fig. 5 and Fig. 6.

Hydrogen bubble generation on the cathode surface is controlled with a constant current source (Gamry Instruments), which simultaneously monitors the applied voltage. During the electrolysis some of the produced hydrogen bubbles stay attached to the cathode and progressively cover its surface.

The pressure distribution near the cathode during vibration is characterized with a needle hydrophone (Müller Plate, Müller Instruments). Pressure field measurements shown in Fig. 1d was obtained by scanning the hydrophone point-by-point on a motorized translation stage across a 1 mm × 1 mm grid at a fixed stand-off distance of approximately 1 mm above the electrode surface in a dedicated ultrasound characterization tank. At each grid position the peak pressure amplitude was recorded, and the resulting spatial map was superimposed on the electrode geometry.

### 2.2 Structural vibration model

**Fig. 3:**
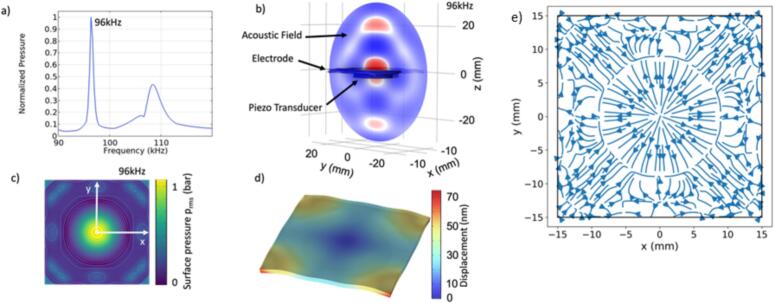
**Structural vibration model:** Finite Element simulation of the acoustic field generated by the vibrating electrode. a) Frequency response of the transducer driver electrode. b) Side view of the acoustic emission into water at the resonance frequency of 96 kHz in the plane x = 0. c) RMS acoustic pressure emitted from the electrode (top view) at 96 kHz and 10 V driving amplitude 0. d) Deformation of the electrode at phase 0 for 96 kHz at 10 V transducer voltage. **Bjerknes force model:** e) Direction of the primary Bjerknes force in the (x,y)-plane for bubbles with a radius of R_0_ = 50 μm driven at p_a_ = 1 bar and a frequency of 96 kHz. Note the dominant attraction of the bubbles towards the circular node with a radius of r = 8 mm with origin (0,0).

The vibrations and acoustic emission from the electrode are modelled with the finite element method (FEM). The model accounts for the electric field in the piezoelectric material (PZT 4), deformation of the piezoelectric transducer and the structural vibrations of the linear elastic plate (Nickel) as well as the linear acoustic radiation into the liquid (water). The simulations are conducted with COMSOL Multiphysics® [35]. The vibrations of the plate are coupled to the acoustic field in the liquid. The electric field of the piezoelectric transducer is calculated from the applied potential and coupled to the plate as a linear piezoelectric material using the stress-charge formulation[36]. To speed up the 3D simulations we make use of the symmetry of the domain. Suitable boundary conditions are used for the coupling between the transducer and the plate and between the plate and the liquid. Details of the model which includes properties of the plate and coupling between the acoustic field and the structural vibrations are available in the supplementary material (SM1). To minimize reflections back into the computational domain, we embed the computational domain in a sphere with boundary condition of spherical wave emission The simulation of the acoustic field generated by the vibrating electrode reveals the frequency response of the electrode (Fig. 3a), the acoustic emission into the liquid at the plate’s resonance frequency of 96 kHz (Fig. 3b), the root-mean-squared (RMS) acoustic pressure emitted from the electrode (Fig. 3c), and the deformation of the electrode which is time and frequency dependent (Fig. 3d).

### 2.3 Bjerknes force model

#### 2.3.1 Primary Bjerknes force model

The primary Bjerknes force $F_{B1}$ is caused by the pressure gradient acting over the oscillating bubble volume, $V_{b}\left(t\right)$*.* It is defined as:

(1) $${\vec{F}}_{B1}\left(\vec{x}\right)=-<V_{b}\left(t\right)\nabla p\left(t,\vec{x}\right){>}_{T}$$

where the brackets <>_T_ denote a time average over one or more complete oscillation periods [37]. The pressure and the pressure gradient are calculated from the finite elements model of the pressure on the surface of the electrode. The bubble dynamics is calculated using the Keller-Miksis model assuming spherical bubble symmetry, that accounts for surface tension, compressibility of the liquid and viscosity; for details we refer the reader to review of Lauterborn and Kurz [37]. This approach neglects the effect of the boundary on the bubble oscillations. This is done for simplicity as the details of the near boundary flow from oscillating and translating bubbles are neither resolved in the experiments nor fully understood in modeling. Yet, previous simulations have revealed that this simplification provides qualitative agreement to experiments [38], [39].

The primary Bjerknes force field in the *x*,*y*-plane can be visualized by plotting the lines tangent to force $F_{B1}$, i.e. the streamlines. For this, we calculate the *x* and *y* components of the force and plot the streamlines for starting positions spaced equidistantly. If the bubbles would only respond to the primary Bjerknes force, then the streamlines would be the trajectories of the bubble. Fig. 3e depicts the resulting streamlines for bubbles with an initial radius $R_{0}=50\;\mu m$ driven at f=96kHz with the pressure calculated from the FEM.

**Fig. 4:**
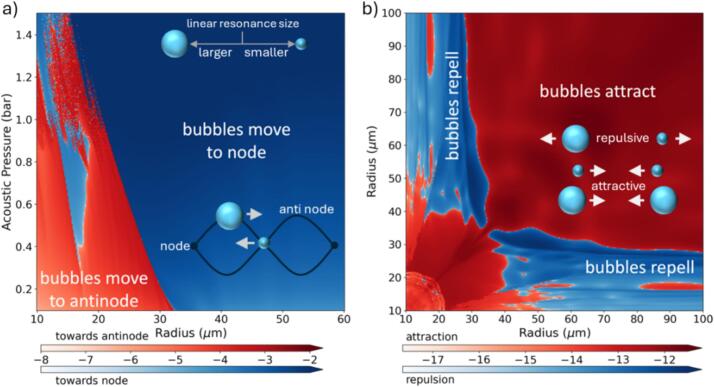
A) primary Bjerknes force and b) secondary Bjerknes force at 96 kHz using the Keller Miksis equation for the bubble dynamics.

The strength and direction of the primary Bjerknes force on a logarithmic scale as a function of bubble radius and pressure amplitude is illustrated in Fig. 4a. For this we omit the gradient of the pressure field and solve instead

(2) $${\vec{F\prime }}_{B1}\left(\vec{x}\right)=-<V_{b}\left(t\right)\;k\;p\left(t\right){>}_{T}$$

where *k* is the wave number and again using the Keller Miksis model [37].

#### 2.3.2 Secondary Bjerknes force model

The secondary Bjerknes force is the result of pressure-based interaction between two nearby oscillating bubbles. It can be written as a scaler quantity $F_{B2}$ that is the force caused by bubble 2 acting on bubble 1, where $\vec{e_{21}}$ is the vector pointing from 2 to 1. The secondary Bjerknes force acting on bubble 1 is

(3) $$F_{B2}^{1}=\vec{F_{B2}^{1}}\cdot \vec{e_{21}}=\frac{\rho }{4\pi d^{2}}<\frac{\text{d}V_{1}\left(t\right)}{\text{d}t}\;\frac{\text{d}V_{2}\left(t\right)}{\text{d}t}{>}_{T}=\frac{1}{d^{2}}\;f_{B2}^{1}$$

where *d* is the distance between the bubbles, $V_{1}\left(t\right)$ and $V_{2}\left(t\right)$ are the volumes of bubble 1 and 2, respectively, and ρ is the density of the liquid. In the bubble dynamics model for bubble 1 one has to account for the pressure emitted from bubble 2 to bubble 1. Details are provided in Ref.[33]. The force constant $f_{B2}^{1}$ on a logarithmic scale for attractive (positive, red) and repulsive (negative, blue) magnitude is shown in Fig. 4b.

## 3 Results

### 3.1 Vibrating plate influence on the hydrogen bubbles

As already mentioned, two acoustic radiation forces act on the bubbles during the electrode oscillation. The primary Bjerknes force is the force the oscillating bubble experience due to a pressure gradient of the acoustic field. The modes (Chladni figure) of the electrode generate an acoustic field that not only drives the volume oscillations of the bubble but also set up a standing wave pattern. It is this pattern that determines the direction of the oscillating bubbles. For linear (small amplitude) bubble oscillations a bubble below resonance size oscillates in phase with the acoustic field and is moving to the pressure node while a bubble above resonance size oscillates out of phase and experiences force to the pressure antinode [40]. The linear resonant bubble size is [32]:

(4) $$R_{r}=\frac{1}{2\pi f_{r}}\sqrt{\left(\frac{3\kappa p_{0}-2\sigma }{\rho }\right)}$$

where $p_{0}$ is the hydrostatic pressure at the equilibrium, ρ is liquid density, σ is liquid surface tension and κ the polytropic exponent of the process (for adiabatic process κ≈1.4). For the excitation frequency of $f_{r}=96\;\;kHz$ the bubble’s resonance radius is approx. 30 μm. However, as the driving pressure from the electrode is large (Fig. 1d), the bubble does not respond as linear oscillator and the non-linear terms of the Keller Miksis equations become important. Fig. 4a depicts the resulting force and direction of the force for non-linear oscillations as a function of bubble size and driving pressure. For small driving pressures, say 0.1 bar, bubbles below the resonance radius are attracted to the pressure antinode and above to the node as expected for linear bubble oscillations. Yet, with increasing acoustic pressure, smaller bubbles become attracted to the node, which is a result of shift of the non-linear resonance radius towards smaller radii (shown by the transition between red and dark blue regions in Fig. 4b). Yet the complex bubble oscillations also lead to islands of reversed attraction. In summary we find that with increasing driving pressures more bubbles become attracted to the pressure nodes, i.e. to regions of low pressure. Fig. 4b depicts the force constant $f_{B2}^{1}$ (eq. (3) on a logarithmic scale for attractive (positive, red) and repulsive (negative, blue) magnitude which further influences the movement of the hydrogen bubbles over the vibrating surface.

### 3.2 Dynamics of a group of bubbles on a vibrating electrode

**Fig. 5:**
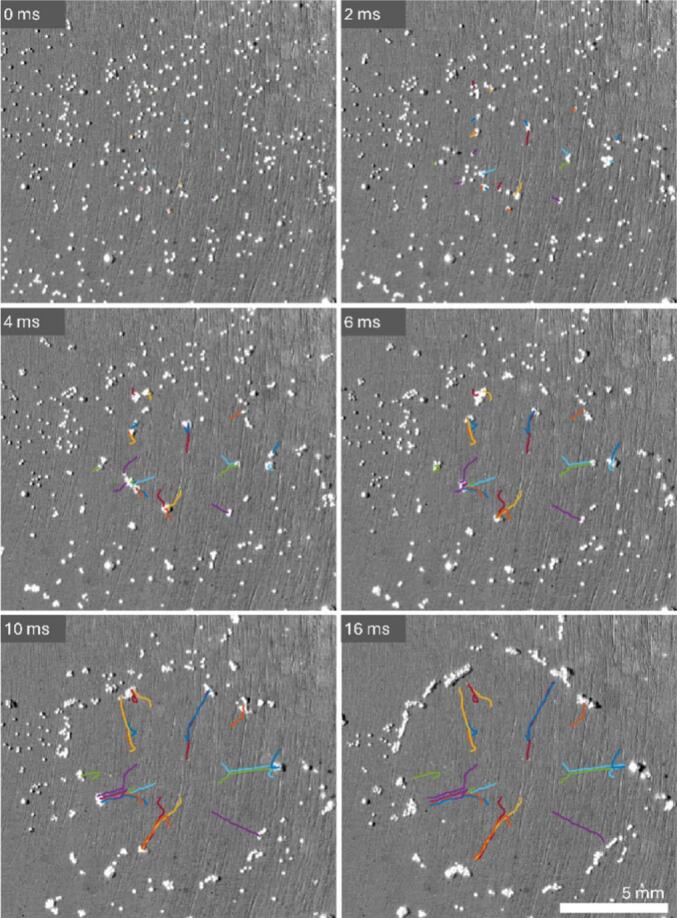
Selected frames from a high-speed video showing the electrode surface after the vibration is turned on. The colored lines mark the selected bubbles during the observation.

When observing the movement of the hydrogen bubbles (Fig. 5 and Video S1), it can be seen, that the bubbles move away from the central area of the cathode but always stay in focus. This indicates that for the duration of the vibration they move in the focal plane parallel to the surface. Increasing the acoustic intensity beyond the levels used here would not repel bubbles from the electrode but would strengthen their migration toward the pressure nodes due to the Bjerknes forces. Fig. 5 presents selected images from the high-speed video (2 kfps) showing the movement of the hydrogen bubbles from the beginning of the vibration until most of the bubbles move towards the vibration nodes and the cathode surface is exposed. Several of these bubbles have a track marked with different colors showing their path from the start of their motion. In some cases, the bubbles first merge and then migrate together towards the node. This initial merging of the bubbles is attributed to the second Bjerknes force, while the movement towards the node is attributed to the first Bjerknes force. Together they contribute to the hydrogen bubble removal from the cathode surface.

**Fig. 6:**
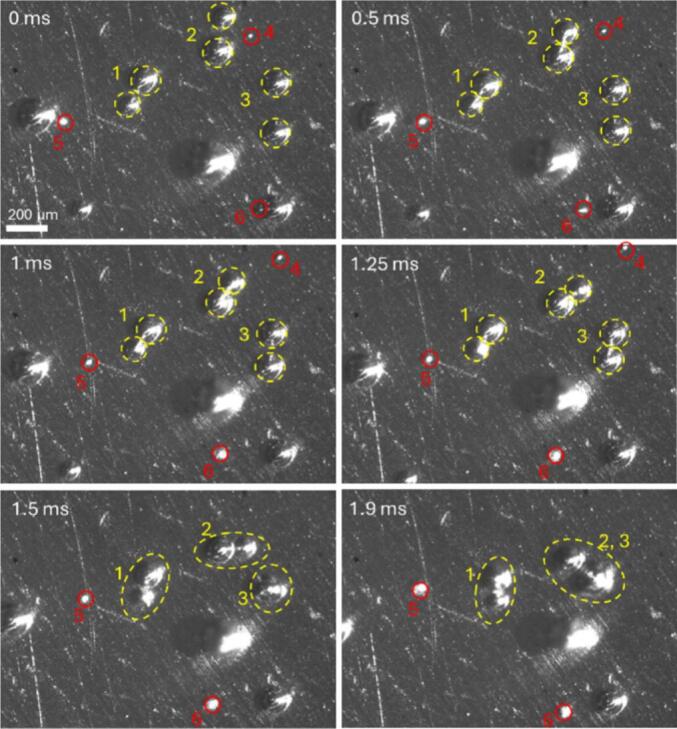
Hydrogen bubble movement on an electrode surface after switching on the vibration. The yellow dashed line marks the interaction of bubbles of similar sizes. The red circles mark the small bubbles that are pushed away from the much larger bubble.

Looking at the surface under higher magnification (Fig. 6) and higher framerate of 20 kfps, we can see the interaction between the hydrogen bubbles. We note that the frame rates of 2 kfps and 20 kfps are chosen to resolve the translational migration of the bubbles, which occurs on the millisecond timescale as a time-averaged response to the 96 kHz acoustic driving. Similar to the previous case, the bubbles are stationary before the electrode vibration is switched on. Thereafter, the bubbles of similar size start to move together due to the attractive secondary Bjerknes force, as shown in Fig. 6 for cases 1, 2 and 3, marked with the yellow dashed line. This force leads to the bubbles grouping up and their coalescence observed for the group of bubbles marked with 2 and 3 at the time of t = 1.9 ms. The repulsive Bjerkness interaction can be observed for the smaller bubbles marked with the red circles, which are close to the larger bubbles before the vibration starts. In this case, the bubbles marked in red are repelled by the larger bubbles. Both interactions contribute to the onset of bubble movement. In addition, the bubbles are affected with the primary Bjerkness force, which begins to pull them towards the nodes.

### 3.3 Reducing bubble induced ohmic losses and efficiency

In order to investigate how the induced vibrations and subsequently the migration of the bubbles to the nodal points affect the reduction in ohmic losses we have recorded the overvoltage. The overvoltage is defined as the difference between the measured cell voltage and the potential of 1.23 V. At the present current density of 11 mA/cm^2^, the total overvoltage of approximately 100 mV includes contributions from activation, electrolyte ohmic drop, and bubble-induced resistance.

**Fig. 7:**
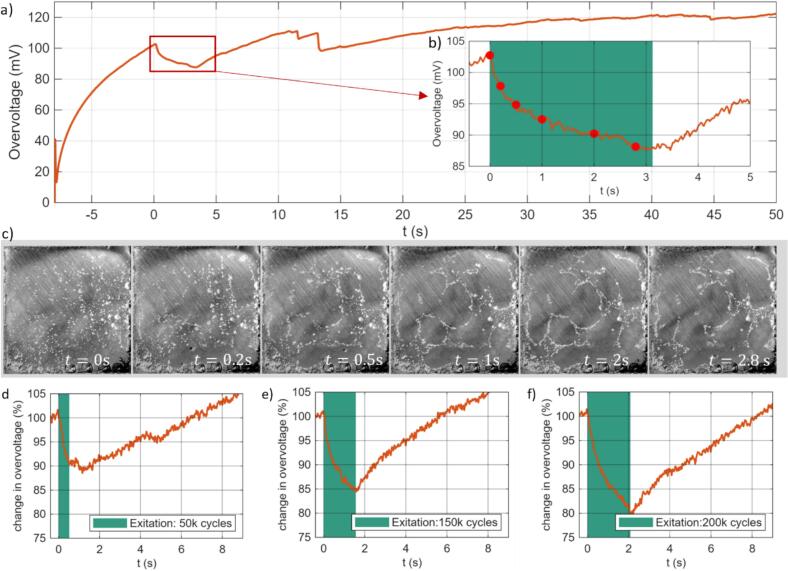
Overvoltage reduction due to the vibration of the electrode: Potentiostat measurement of the cell overvoltage change (orange) after the electrolytic cell is turned on (green areas), b) zoomed into the region where the vibration was turned on (green) with the red dots marking the time of the frames from the high-speed video of the electrode surface shown in c). In panels d)–f) the overvoltage is normalized to its value at the onset of vibration and expressed as a percentage to enable direct comparison of the relative reduction across different excitation d) 0.52 s, e) 1.65 s and f) 2.08 s.

Fig. 7a,b illustrates this overvoltage in combination with the hydrogen bubble coverage, of the electrode plotted in Fig. 7c. Once the electrolytic cell is turned on, i.e. current is fixed to 100 mA, the overvoltage increases (marked in orange). Once the overvoltage reaches 100 mV the piezo vibrating the cathode is switched on (assigned as our time reference *t* = 0), for a set duration of 3.2 s (marked in green), see Fig. 7b. In this and the following experiments the piezo was driven at the same voltage amplitude of 110 V. The corresponding frames from the high-speed camera recorded at the times marked with a red symbol in Fig. 7b are shown in Fig. 7c. The voltage drop occurs within the first 100 ms, which is related to the movement of the bubbles to the nodal regions of the vibrating cathode. After that, the falling trend in the overvoltage value slows down which is attributed to the fact that the majority of the larger bubbles have moved to the nodal regions. This is confirmed from the selected frames shown in Fig. 7c. After the end of the electrode vibration, i.e. at *t* = 3.2 s, the overvoltage increases again slowly as the cathode surface is blocked with the production of newly formed hydrogen bubbles.

This observation suggests that pulsed operation of the piezo may be favorable as compared to continuous vibration of the plate. Therefore, we compare in Fig. 7d-f the overvoltage drop for different durations of electrode vibrations. We find that already 50,000 cycles or about 0.52 s result in a reduction of the overvoltage for 6 s. The overvoltage is reduced more for longer durations, yet it has little effect on the duration of the voltage reduction, see Fig. 7d-f.

At the present operating conditions (100 mA, approximately 100 mV overvoltage), the excess power dissipated due to bubble‐induced ohmic losses is approximately 10 mW. In this case the acoustic excitation reduces the overvoltage by roughly 10 %, yielding a saving of about 1 mW. The electrical input to the piezoelectric transducer is on the order of several hundred milliwatts, which exceeds the present ohmic saving. However, the current experimental setup is optimized for optical access and bubble tracking rather than overall energy efficiency. At industrially relevant current densities (200–500 mA/cm^2^), a denser bubble coverage and higher ohmic losses are expected, and the energy balance of an applied acoustic excitation would become more favorable.

## 4 Conclusion

We have demonstrated an active method for hydrogen bubble removal through ultrasonic excitation applied directly at the electrode, resulting in the clearing of the active electrode surface and a significant reduction in ohmic losses. A comprehensive experimental investigation was conducted using high-speed imaging of the cathode surface, pressure field measurements near the electrode, and overvoltage monitoring to characterize how cathode vibration influence the electrolytic process.

Hydrogen bubbles were generated under constant current conditions, progressively covering the cathode surface during electrolysis. Upon activating the piezoelectric element at the excitation frequency of 96 kHz, the bubbles detach and migrated across the surface. After a transient period, a distinct spatial pattern emerged, corresponding to the vibrational modes of the flat plate electrode. The underlying mechanism involves acoustic fields generated by the electrode's vibrational modes, which simultaneously drive bubble volume oscillations and establish a standing wave pattern. This acoustic field governs both the directional migration of oscillating bubbles on the electrode surface and the forces acting upon them, specifically the primary and secondary Bjerknes forces.

Bubble removal was found to be dominated by the primary Bjerknes force, which arises from the pressure gradient in the acoustic field acting on the oscillating bubble. As the driving pressure amplitude was comparable to the internal gas pressure, bubble oscillations entered the nonlinear regime. The Keller-Miksis model was employed to predict bubble dynamics and migration direction, revealing that increasing driving pressures cause more hydrogen bubbles to migrate toward pressure nodes, i.e., regions of minimum acoustic pressure. This prediction was confirmed by experimental observations.

Beyond bubble migration dynamics, we demonstrated that electrode vibrations and the subsequent bubble redistribution to nodal regions significantly reduce ohmic losses. The correlation between overvoltage drop and hydrogen bubble coverage of the electrode surface showed that the most rapid voltage reduction occurred within approximately 100 ms, corresponding to the timescale of bubble migration to the nodal regions. Operating the electrode in a pulsed mode may have advantages in terms of efficiency, i.e. the reduction of ohmic losses as compared to the costs of driving the electrode. For instance, a 2 s vibration period reduced the overvoltage by 20 %, with the voltage requiring 7 s to return to its initial value after deactivation.

The demonstrated method of vibrational modes to introducing acoustic fields on bubble-forming surfaces may be applicable beyond electrolysis. In boiling applications [41], this approach could prevent vapor bubbles from inhibiting heat transfer by covering the heater surface, thereby mitigating the boiling crisis phenomenon [42].

## CRediT authorship contribution statement

**Vid Agrež:** Writing – review & editing, Writing – original draft, Visualization, Validation, Methodology, Investigation, Formal analysis, Data curation, Conceptualization. **Zeinab Heidary:** Writing – review & editing, Visualization, Investigation, Conceptualization. **Manolis Gavaises:** Writing – review & editing, Methodology, Conceptualization. **Rok Petkovšek:** Writing – review & editing, Validation, Supervision, Resources, Project administration, Funding acquisition, Formal analysis, Conceptualization. **Claus-Dieter Ohl:** Writing – review & editing, Writing – original draft, Visualization, Validation, Supervision, Resources, Project administration, Methodology, Investigation, Funding acquisition, Formal analysis, Data curation, Conceptualization.

## Declaration of competing interest

The authors declare that they have no known competing financial interests or personal relationships that could have appeared to influence the work reported in this paper.

## Appendix A . Supplementary data

Supplementary data include SM1 Supplementary Material file describing details on the finite element simulations and video S1 corresponding to the Fig. 5.
