# Supplementary material for: Chladni figures reduce ohmic losses in alkaline electrolysis
Source: Ultrason Sonochem. 2026 Mar 22;128:107833. doi: 10.1016/j.ultsonch.2026.107833 (PMC13050082; doi:10.1016/j.ultsonch.2026.107833)
Supplement: Supplementary Data 1 [file mmc1.docx]

**Supplementary Material (SM1) for
“Chladni figures reduce ohmic losses in alkaline electrolysis”**

Vid Agrež^1,2^, Zeinab Heidary^1^, Manolis Gavaises^3,4^, Rok Petkovšek^2^, Claus-Dieter Ohl^1*^

*^1^Faculty of Natural Sciences, Institute for Physics, Otto-von-Guericke-University Magdeburg, Universitätsplatz 2, 39106 Magdeburg, Germany*

*^2^Faculty of Mechanical Engineering, University of Ljubljana, Aškerčeva 6, SI-1000 Ljubljana, Slovenia*

*^3^Faculty of Process Engineering, Otto-von-Guericke-University Magdeburg, Universitätsplatz 2, 39106 Magdeburg, Germany*

*^4^School of Science & Technology, City, University of London, Northampton Square, EC1V 0HB, London, United Kingdom*

***Details on the finite element simulations***

The finite element simulations utilize the multiphysics package COMSOL. The domain is shown in Fig. S1 and consists of electrostatics (es), pressure acoustics (acpr), and solid mechanics (solid) solvers. The geometry consists of a spherical acoustic domain, the electrode that is modelled as a linear elastic solid, and the piezo, that is modeled as a piezoelectric solid coupled to the electrostatic solver. We utilize the symmetry of the 3-dimensional problem to reduced computational time. The results in the paper are presented by mirroring the domain shown in Fig. S1 three times successively, at the planes with normal vectors (-1,1,0), (0,1,0), and (1,0,0). Then the electrode is a plate with 30x30mm^2^ and 1mm thickness. The piezo is disk with 2mm thickness and 20mm diameter, and the acoustic domain has a diameter of 60mm.


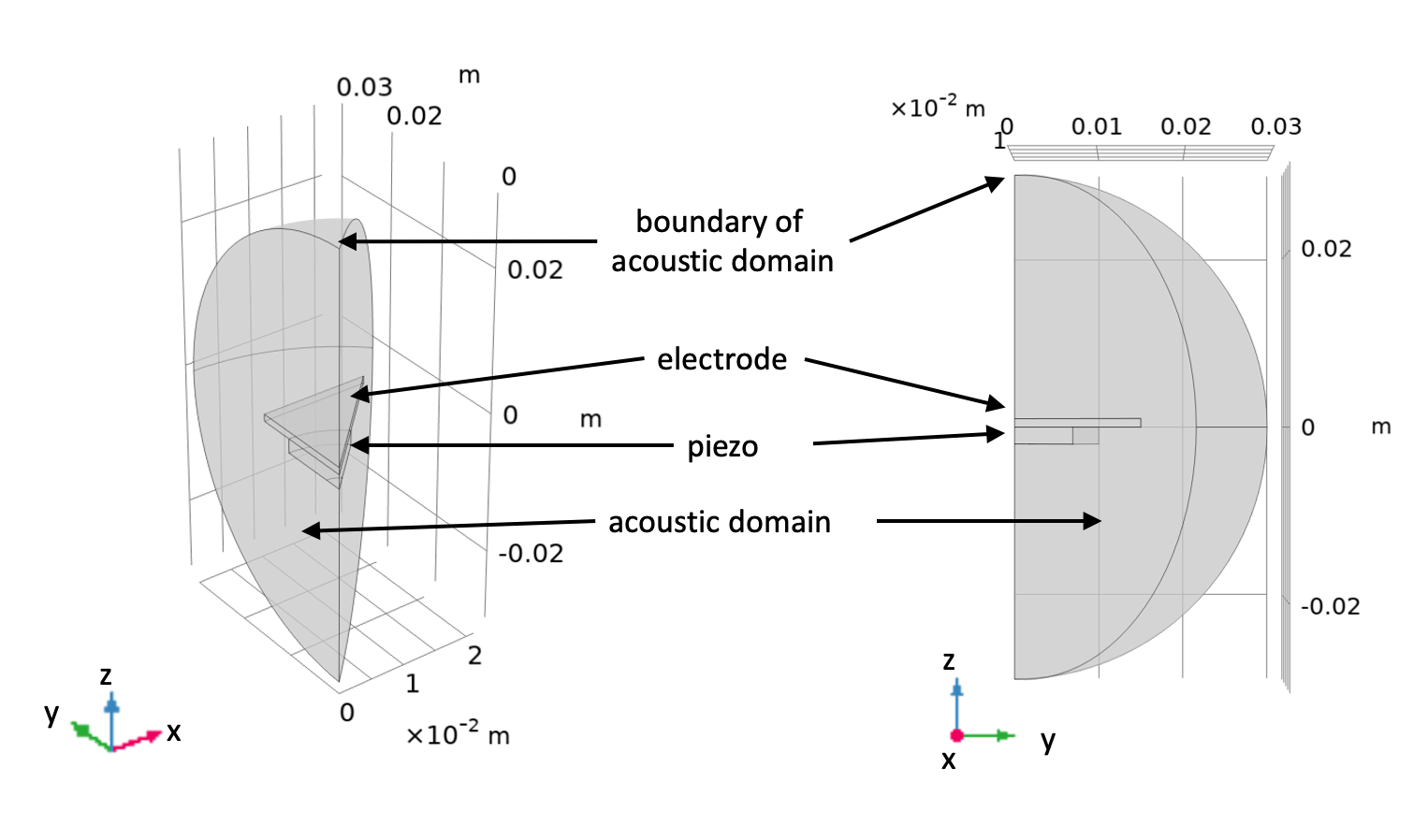


*Figure S1: Domain of the finite element simulation.*

The boundary condition at spherical acoustic domain surface is spherical wave radiation. For the solid the structure (electrode and piezo) is coupled to the acoustic domain through Eq. (1).

 (1)

Here, **u** is the displacement of the electrode and *p* the acoustic pressure. Additionally, we implement coupling of the piezoelectric response with the solid deformation through the stress-charge formulation [1]. There is a small central region of the piezo of 5 mm diameter, that is fixed, i.e. **u**=**0**.

The acoustic domain contains water with a density of ρ_w_=998 kg/m^3^, and a speed of sound of *c*=1481 m/as. The piezo is modeled as a PZT-4 material with the default values from COMSOL, and the electrode is modeled as linear elastic material with ρ_E_=8908 kg/m^3^, elastic modulus of *E*=200GPa, and a Poisson ratio ν=0.3.

While there exists a simplified model for thin plates [2] that allows an approximate derivation of the modes and shape, here we can’t assume a thin plate and therefore the full equations for linear elastic solids are solved, i.e. the Cauchy momentum equation (Newton’s second law), the strain-displacement equations, and the linear elastic constitutive equations (Hook’s law) relating stress and strain. Details are available in most textbooks on elasticity, e.g. Ref.[3].

All equations are solved in the frequency domain.

[1] COMSOL AB, Structural Mechanics Module User’s Guide, in: COMSOL Multiphysics® v. 6.4, COMSOL AB, Stockholm, Sweden, 2025: pp. 209–230.

[2] S.V. Bosakov, Eigenfrequencies and modified eigenmodes of a rectangular plate with free edges,

J. Appl. Math. and Mech., 73, 2009, 688-691.

[3] Sadd, Martin H. 2020. *Elasticity: Theory, Applications, and Modeling*. 4th ed. Academic Press.
